# Establishment of the 1^st ^World Health Organization International Standard for *Plasmodium falciparum *DNA for nucleic acid amplification technique (NAT)-based assays

**DOI:** 10.1186/1475-2875-7-139

**Published:** 2008-07-24

**Authors:** David J Padley, Alan B Heath, Colin Sutherland, Peter L Chiodini, Sally A Baylis, the Collaborative Study Group

**Affiliations:** 1 National Institute for Biological Standards and Control, Blanche Lane, South Mimms, Potters Bar, Hertfordshire EN6 3QG, UK; 2 Hospital for Tropical Disease, Mortimer Market, Off Tottenham Court Road, London, WC1E 6AU, UK

## Abstract

**Background:**

In order to harmonize results for the detection and quantification of *Plasmodium falciparum *DNA by nucleic acid amplification technique (NAT)-based assays, a World Health Organization (WHO) collaborative study was performed, evaluating a series of candidate standard preparations.

**Methods:**

Fourteen laboratories from 10 different countries participated in the collaborative study. Four candidate preparations based upon blood samples parasitaemic for *P. falciparum *were evaluated in the study. Sample AA was lyophilized, whilst samples BB, CC and DD were liquid/frozen preparations. The candidate standards were tested by each laboratory at a range of dilutions in four independent assays, using both qualitative and quantitative NAT-based assays. The results were collated and analysed statistically.

**Results:**

Twenty sets of data were returned from the participating laboratories and used to determine the mean *P. falciparum *DNA content for each sample. The mean log_10 _"equivalents"/ml were 8.51 for sample AA, 8.45 for sample BB, 8.35 for sample CC, and 5.51 for sample DD. The freeze-dried preparation AA, was examined by accelerated thermal degradation studies and found to be highly stable.

**Conclusion:**

On the basis of the collaborative study, the freeze-dried material, AA (NIBSC code No. 04/176) was established as the 1^st ^WHO International Standard for *P. falciparum *DNA NAT-based assays and has been assigned a potency of 10^9 ^International Units (IU) per ml. Each vial contains 5 × 10^8 ^IU, equivalent to 0.5 ml of material after reconstitution.

## Background

Each year there are an estimated 300 million acute cases of malaria worldwide, accounting for more than one million deaths annually [1]. In humans, malaria is caused by infection with five species of *Plasmodium *(*Plasmodium falciparum*, *Plasmodium malariae, Plasmodium ovale*, *Plasmodium vivax *and *Plasmodium knowlesi*). *Plasmodium falciparum *is associated with the greatest morbidity and mortality, accounting for approximately 95% of deaths due to malaria [2]. Although most of these cases are found in endemic tropical regions of Africa, Asia, Central and South America, cases of imported malaria are reported in non-endemic areas. In Europe it has been estimated that there are in excess of 10,000 cases of imported malaria per year, with around 2,000 cases being recorded annually in the UK alone [3,4]. Estimates by the Centers for Disease Control and Prevention (United States) have indicated that there were approximately 1,528 cases, including seven fatal cases, of imported malaria in the United States in 2005, largely due to travellers, immigrants and servicemen returning from malaria endemic regions [5]. Transfusion transmitted cases of malaria can also occur, by the use of contaminated blood, and may result in significant disease or even death in the recipients, particularly when *P. falciparum *is the causative agent [6].

Malaria diagnosis has relied upon microscopic analysis of Wright's or Wright-Giemsa stained blood smears to detect infection and determine parasite loads [7]. Slide interpretation requires considerable expertise and is difficult to standardize, particularly at low levels of parasitaemia or where mixed species infections occur [8,9]. Nucleic acid amplification technique (NAT)-based assays, such as PCR are becoming increasingly employed in the diagnosis of malaria [10,11]. Studies have shown that PCR can improve sensitivity and species discrimination, when compared to either microscopy or serological methods [12]. Although numerous PCR methods have been developed for the laboratory diagnosis and clinical management of malaria, the reported sensitivities of these assays vary widely. These differences may be a result of intrinsic variability in assay sensitivity or a consequence of calibration using different reference reagents, which are poorly standardized. In terms of quality assurance, it is of concern that variability in the implementation of assays between laboratories could give different results for the same patient sample.

In the last ten years, a number of International Standards have been developed by the World Health Organization (WHO) for NAT-based assays. The first was established in 1997 for hepatitis C virus (HCV) RNA [13]. This standard was pivotal in the introduction of HCV RNA NAT screening of blood and plasma donations, used in transfusion and the manufacture of plasma derived medicinal products, where previously there had been transmissions of HCV. Further standards have been established for hepatitis B virus (HBV) DNA, HIV-1 RNA, parvovirus B19 DNA, hepatitis A virus (HAV) RNA [14-17]. The standards have allowed the development of regulatory requirements for the detection of these blood borne viruses in plasma, by setting commonly recognized thresholds and limits of detection. The standards have been used in the calibration of secondary standards and working reagents, and have been used in the validation of assays for both the qualitative and quantitative NAT-based techniques. These blood-borne virus standards have also found use in the clinical setting, both for diagnosis and for monitoring viral loads in response to antiviral therapy. Moreover, results in a number of commercially available assays for blood borne viruses are expressed in terms of WHO International Units associated with the respective standards.

This paper describes a collaborative study to evaluate four candidate standards for their suitability for use as a WHO International Standard for *P. falciparum *DNA for use in NAT-based assays. The value of such a standard will be to ensure consistent assay implementation between laboratories; allow inter-laboratory comparisons; provide materials for assay validation; and in particular to generate secondary standards to monitor routine assay use. The need for such a material was recognized by the WHO Expert Committee on Biological Standardization (ECBS) in 2004.

## Methods

### Candidate standards

Four candidate standards were included in this study. Sample AA was a freeze-dried preparation of blood, obtained from a patient infected with *P. falciparum *following an exchange transfusion. Local Research Ethical Committee approval was obtained at the London Hospital for Tropical Diseases for the collection and subsequent use of this sample. The blood used to prepare sample AA, was determined to have an overall parasitaemia of approximately 9.8% by light microscopy. Sample AA was stored at -70°C until lyophilization. The conditions for lyophilization were as previously described [13,14]. Lyophilized vials of AA have been stored at -20°C with constant temperature monitoring, at the National Institute for Biological Standards and Control (NIBSC). Sample BB was a liquid preparation, of *in vitro *cultured *P. falciparum *(3D7 strain) prepared in leucodepleted blood, with a parasitaemia of approximately 10%. Sample CC was a liquid preparation of approximately 6.9% parasitaemia of *P. falciparum *infected blood, obtained from the same patient as sample AA. Sample DD was a 1:1000 dilution of sample CC prepared in leucodepleted blood. Samples BB, CC and DD were dispensed in 0.5 ml volumes and stored as liquid/frozen preparations at -70°C. All samples tested negative by PCR for the following viral markers: HBV DNA, HCV RNA, HAV RNA, HIV-1 RNA and parvovirus B19 DNA.

### Design of the study

Fourteen laboratories participated in the collaborative study. Laboratories were sent four vials of each of the candidate standards. The participants were requested to store samples at or below -70°C until analysis. The lyophilized preparation (AA) was reconstituted using 0.5 ml of nuclease-free deionized water immediately prior to analysis, with gentle agitation for 20 minutes to fully dissolve the contents. The liquid preparations (BB, CC and DD) were to be thawed quickly before use. Participants were requested to test the panel of candidate standards in four independent assays for *P. falciparum *DNA, using fresh vials of the four candidates for each assay run. In the case of qualitative assays, serial dilutions of the samples were analysed in four independent assays. In the first qualitative assay, ten-fold dilutions were performed to determine the end point for the detection of *P. falciparum *DNA. In each of the subsequent three assays, a minimum of two half-log_10 _(i.e. 1:3.2) dilutions either side of the pre-determined end-point were assayed, and results reported as positive or negative. Participants were requested to prepare dilutions in the sample diluent normally used in their assay system. In the case of quantitative assays for *P. falciparum *DNA, results were used directly as returned by laboratories, after correction for any dilutions made.

### Statistical methods

The qualitative end-point assays were analyzed using the Poisson model, as previously described for collaborative studies for other NAT standards [13,15]. All estimates were expressed as log_10 _PCR detectable units/ml. Overall means were calculated as arithmetic means of the log_10 _estimates.

### Stability studies

The lyophilized candidate standard (AA) was analysed for its stability over time in accelerated thermal degradation studies. Samples were incubated in temperature controlled environments, withdrawn at specified times and analysed for *P. falciparum *DNA content as described below. Following reconstitution, 200 μl volumes of sample for AA were extracted using the MagNA Pure LC instrument with software version 4.0 (Roche Applied Science, Mannheim, Germany). Samples were extracted using the DNA Isolation Kit I (Roche Applied Science, Mannheim, Germany) according to the manufacturer's instructions. Elution was performed with 100 μl of elution buffer. Real-time PCRs were performed on the LightCycler 2.0 instrument (Roche Applied Science, Mannheim, Germany). An in-house TaqMan assay was performed using primers selected from the most conserved regions of the 18S ribosomal RNA (rRNA) gene. The primer sequences were as follows: forward primer, 5' CAG ATG TCA GAG GTC AAA TTC TAA GAT T 3'; reverse primer, 5' TCC CTT AAC TTT CGT TCT TGA TTA ATG 3'. The sequence of the fluorogenic hydrolysis probe was as follows: 5' (FAM) CTG GAG ACG GAC TAC TGC GAA AGC ATT TG (TAMRA) 3'. Amplification reactions were performed using the LightCycler FastStart DNA Master Hybprobe kit (Roche Applied Science, Mannheim, Germany). The amplification conditions were as follows: 95°C for 10 min, then 45 cycles of the following sequential steps: 95 °C for 15s, 60°C for 1 min. Fluorescence data was collected during the combined annealing/extension step and detected at 530 nm. A standard curve was generated using serial ten-fold dilutions of a sample with a concentration of 180,000 parasites per μl, as determined by Giemsa-stained thin film microscopy by a proficient operator, with independent confirmation.

## Results

### Data returned and data analysis

Fourteen laboratories from 10 countries, returned results from four assays for each of the four separate preparations. The participants included parasitology laboratories from tropical medicine institutes, universities, hospitals, quality control laboratories, manufacturers of diagnostic kits and other laboratories. The majority of assays used by the participating laboratories were ones developed in-house, although participants returned results from commercial assays. Throughout this study, a code number has been allocated at random for each laboratory and does not necessarily represent the order of the participants described in Table 1. The types of assays used by participants are listed in Table 2.

**Table 1:** Collaborative study participants

Name	Affiliation
Dr A Calderaro	University of Parma, Parma, Italy
Prof. PL Chiodini	Hospital for Tropical Diseases, London, UK
Dr C Defer	EFS Nord de France, Lille, France
Dr I Felger	Swiss Tropical Institute, Basel, Switzerland
Dr KC Kain	Center for Travel and Tropical Medicine, Toronto, Canada
Prof. S Krishna	St. George's Hospital, London, UK
Dr R Lee	Institute of Clinical Pathology & Medical Research, Westmead, Australia
Mr DJ Padley	NIBSC, South Mimms, UK
Dr F Perandin	University of Brescia, Brescia, Italy
Dr G Pisani	Istituto Superiore di Sanità, Rome, Italy
Dr T Ruckes	Qiagen GmbH, Hamburg, Germany
Dr AJ da Silva	Centers for Disease Control and Prevention, Atlanta, USA
Dr S Salueda	Banc de Sang I Teixits, Barcelona, Spain
Prof. R Sauerwein	Radboud University Nijmegen Medical Center, Nijmegen, The Netherlands

**Table 2:** Assay methodologies used by study participants

**Lab No**.	**Nucleic Acid Extraction**	**Nucleic Acid Amplification**	**Sequence Targeted**
1A	QIAamp DNA Mini Blood Kit	Qiagen Taq DNA polymerase	Small subunit rRNA^a^
1B	QIAamp DNA Mini Blood Kit	TaqMan^® ^universal Master MIX	Conserved ATS domain of var genes
2A	QIAamp DNA Blood kit	Eppendorf Master Mix 2.5 × kit, gel based assay	18S rRNA^b^
2B	QIAamp DNA Blood kit	QuantiTectProbe PCR Kit, real-time PCR	18S rRNA^b^
3A	MagNA Pure LC Total Nucleic Acid Isolation Kit	LightCycler Fast Start DNA Master SYBRGreen I	18S rRNA
3B	MagNA Pure LC Total Nucleic Acid Isolation Kit	RealArt™ Malaria LC PCR Kit	18S rRNA
4	QIAamp DNA Blood Mini Kit	MBI Taq DNA polymerase	Small subunit rRNA gene^a^
5	Boom *et al*., 1990 [20]	RNA T7 Polymerase, AMV-RT, RNAse H, bioMerieux	18S rRNA^c^
6	MagNA Pure LC DNA Isolation Kit I	LightCycler FastStart DNA Master^PLUS ^Hybprobe	18S rRNA
7	QIAamp DNA Mini Kit	Taqman 2 × Universal PCR Mastermix	β-tubulin^d^
8	QIAamp DNA Mini Kit	TaqMan^® ^Universal Master Mix	18S rRNA^b^
9	QIAamp DNA Mini Blood Kit	N/A	18S rRNA
10A	QIAamp DNA Mini Blood Kit	Bioline Taq Polymerase	18S rRNA^a^
10B	QIAamp DNA Mini Blood Kit	Fast Start DNA Sybr Green Kit	18S rRNA
11A	Roche High Pure PCR Template Preparation Kit	Applera Taq Polymerase, Applied Biosystems	18S rRNA^e^
11B	Roche High Pure PCR Template Preparation Kit	2 × Taqman Universal	18S rRNA^a^
12	QIAamp DNA Mini Blood Kit	AmpliTaq Gold DNA Polymerase	18S rRNA^a^
13	QIAamp DNA Blood/Mini Kit	artus Malaria PCR Kit	18S rRNA
14	High Pure PCR Template Preparation Kit	Applied Biosystems Taq Polymerase	18S rRNA^e^

Assays performed according to the following published methods ^a^[18,19], ^b ^[extraction method only, [20]], ^c^[21], ^d^[22], ^e^[23], For other assays there were either no previously published methods or the information was not disclosed

Participating laboratories were requested to test the samples in qualitative or quantitative format in order to determine the *P. falciparum *DNA content for each of the four candidate standards. The majority of laboratories provided data from qualitative end-point dilution assays. These laboratories reported the number of samples positive out of the number tested at various dilutions. These were treated as a dilution series and used to provide a single estimate of "PCR-detectable units/ml" in the undiluted sample using the method of maximum likelihood for "dilution assays" as described for the analysis of other NAT standards [13,15]. The model assumes that the probability of a positive result at a given dilution follows a Poisson distribution (with the mean given by the expected number of "copies" in the sample tested, and that a single copy will lead to a positive result). Calculations were carried out using the statistical package GLIM [13,15].

Where a laboratory marked a result as uncertain or +/-, it was treated as negative. Where a laboratory performed more than one assay method, the results were analyzed separately, treated as if from separate laboratories, and coded as for example as laboratories 1A and 1B. Laboratory 1B did not continue the dilution series for sample AA until negative responses were observed. It was therefore not possible to calculate an estimate of units/ml for this sample (see below). For assays 2–4, Laboratory 9 only tested single log dilutions to the endpoint rather than half-log dilutions at the supposed end point. It was still possible to determine estimates of detectable units/ml, but they may be less precise than for other laboratories. Laboratory 10 (10A & 10B) had some positives at very high dilutions. Because the Poisson model could not be fitted with these results, they were excluded as potential false positives. Laboratory 11 (11A & 11B) reported "dilutions not well executed" for assay 2, which was therefore excluded. It was not possible to obtain an estimate of detectable units/ml for sample CC for 11A, as all results were positive. Some of the remaining results from 11B were also inconsistent. Laboratory 14B had some inconsistent negatives at 10^-1 ^and 10^-2 ^for sample DD in assay 2, which were excluded.

Two laboratories provided quantitative results. Laboratory 3B provided quantitative results using a commercial kit (Artus RealArt Malaria LC PCR kit, Qiagen, Hamburg, Germany) for a range of dilutions for each sample. The results were quoted as copies/μl. Laboratory 5, provided results from an in-house quantitative assay, giving results as a mean log_10 _Par/ml based on two sets of triplicate determinations for each sample.

### Estimates of PCR-detectable units/ml

**Figure 1:**
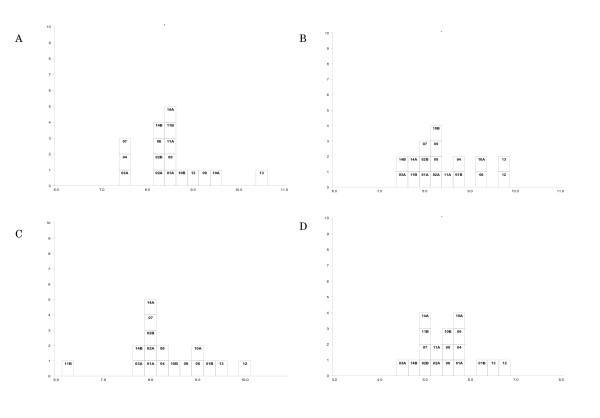
**Histograms of laboratory estimates of polymerase chain reaction (PCR)-detectable units/ml (log_10_) for samples AA, BB, CC and DD (panels A, B, C and D respectively).** The laboratory code numbers are inicated in each box in the respective histograms.

The estimated log_10 _PCR-detectable units/ml for each of the four candidate standards from the qualitative end-point assays are shown in histogram form in Figure 1 and in Table 3. Each box represents the estimate from one laboratory, and is labeled with the laboratory code number. The overall means, range and between laboratory standard deviations are shown in Table 4. The agreement between laboratories is similar to that observed in the collaborative study to establish the 1^st ^International Standard for HCV RNA for NAT-based assays [13]. The quantitative results from laboratories 3B and 5 are shown in Table 5. For laboratory 3B, the results are based on the neat and 1 in 10 dilutions only, as the results did not appear linear for dilutions beyond this. Results were expressed as "copies/ul". For laboratory 5, results were expressed as "Par/ml". The estimates from laboratory 3B are much higher than the results from the qualitative assays, particularly for samples AA, BB and CC. For laboratory 5, the estimates for samples AA, CC and DD are much lower than those from the qualitative assays. The estimate for sample BB is of the same order as the qualitative assays, but 4 log_10 _higher than the equivalent estimates of AA and CC. This may be due to the fact that this particular assay is amplifying RNA and not DNA, compared to the other assays. Samples AA, CC and DD were subject to multiple rounds of freeze-thawing during their preparation, and due to cell lysis, the labile nature of RNA and its susceptibility to degradation by nucleases have resulted in loss of potency of these preparations when RNA levels are examined. Sample BB only underwent a single freeze-thaw cycle before dispatch to study participants and hence has a higher potency in this particular assay for Laboratory 5. These results emphasize that this candidate standard is not suitable for RNA assays.

**Table 3:** Estimated PCR-detectable units/ml (log_10_) calculated from end-point dilution assays.

Lab	Sample			
	
	AA	BB	CC	DD
1A	8.51	8.12	7.96	5.82
1B	-	8.87	9.26	6.23
2A	8.26	8.26	7.98	5.15
2B	8.14	7.90	7.98	5.12
3A	7.44	7.62	7.84	4.51
4	7.56	8.71	8.25	5.84
6	8.14	9.32	8.95	5.62
7	7.54	8.12	7.89	4.97
8	8.44	8.28	8.28	5.58
9	9.31	8.18	8.80	5.80
10A	9.41	9.23	8.99	5.73
10B	8.65	8.36	8.39	5.42
11A	8.46	8.44	-	5.17
11B	8.45	7.70	6.35	5.12
12	8.96	9.64	9.92	6.86
13	10.46	9.85	9.47	6.39
14A	8.62	7.79	8.00	5.07
14B	8.28	7.62	7.68	4.74

Mean	8.51	8.45	8.35	5.55

**Table 4:** Overall mean estimated PCR detectable units/ml (log_10_) from end-point assays

Sample	N	Mean	Min	Max	Range	SD
AA	17	8.51	7.44	10.46	3.01	0.74
BB	18	8.45	7.62	9.85	2.23	0.69
CC	17	8.35	6.35	9.92	3.57	0.83
DD	18	5.51	4.51	6.86	2.36	0.60

N – Number of laboratory estimates

SD – Standard Deviation of log_10 _estimates across laboratories

**Table 5:** Mean estimates from quantitative assays

	Sample			
	
	AA	BB	CC	DD
Lab 3B log_10 _copies/μl	9.93	9.34	10.20	3.48
Lab 5 log_10 _Par/ml	4.21	8.12	3.99	0.93

**Figure 2:**
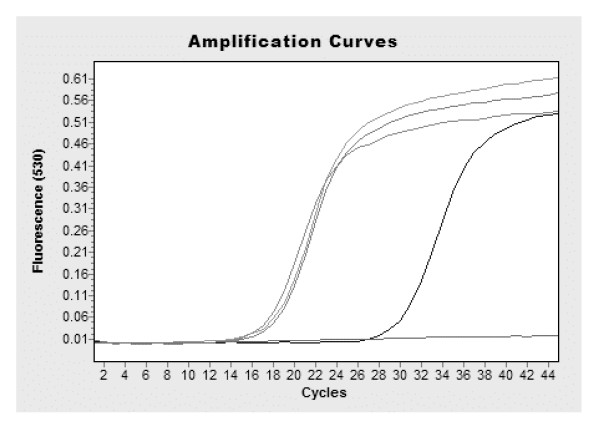
**Amplification plot of samples AA, BB, CC and DD using a TaqMan assay targeting the 18S rRNA gene of *P. falciparum*, as described in Stability Section of the Materials and Methods.** The different samples are indicated on the figure. No template control (NTC).

Figure 2 shows the performance of the four candidate standard preparations in a real-time TaqMan PCR assay (as described in Stability Studies section of the Materials and Methods), which further demonstrates mean estimated PCR detectable units/ml values shown in Table 4. Near identical crossing cycle (Ct) values are found for samples AA, BB and CC, with sample DD generating a much later Ct values reflecting the dilution of this material when it was originally prepared. It should be noted that sample AA was derived from blood from an exchange transfusion that was determined to have an overall parasitaemia of approximately 9.8% which had been determined by light microscopy. Sample BB was a liquid preparation of *in vitro *cultured *P. falciparum *(3D7 strain) with a parasitaemia of approximately 10% was also evaluated in this study. The mean estimates of these two preparations AA and BB are 8.51 and 8.45 log_10 _PCR detectable units/ml respectively based upon the results of the end point dilution assays, demonstrating that the patient derived sample AA gives results that are commutable to the *in vitro *propagated sample derived from the 3D7 laboratory strain of *P. falciparum*.

### Stability studies on candidate sample AA

The stability of sample AA was examined over time in accelerated degradation studies. Samples were incubated in temperature controlled environments, withdrawn at specified times and tested for *P. falciparum *DNA content. A baseline titre was determined by comparison to vials of sample AA stored at -20°C (i.e. the long term storage temperature for lyophilized standards). The overall geometric means (log_10_) for the estimated parasites/ul were determined for the sample AA at each of the different incubation temperatures. This is displayed as the log_10 _drop (Table 6). At the higher temperatures there is clearly some degradation, with a drop of around 0.3 log_10 _after a year at +20°C, and around 0.6 log_10 _at higher temperatures. There was little observed loss at +4°C however, with an apparent increase at 8 months, and a loss of 0.06 log_10 _at 12 months. This data is based on a limited number of vials however, and these differences are within the expected assay variability. If the Arrhenius model for accelerated degradation [24,25] is applied to this data, a predicted loss per year at -20°C of around 20%, or 0.1 log_10 _is obtained. However this is not a precise estimate. This prediction does not appear to be consistent with the observed data for the sample stored at +4°C, which has not shown degradation of the order of that predicted for the -20°C sample. It is not clear whether the Arrhenius model, which is based on a simple first order degradation process, is appropriate for material of this type. Although there is degradation at higher temperatures, there is little observed degradation after 12 months at +4°C and this observed stability is in line with that observed for the WHO International Standards for the blood borne viruses [13,15,17]. Studies of real-time stability are of sample AA are ongoing.

**Table 6:** Stability Study – Loss relative to -20°C Sample (Log_10 _drop)

Months Storage	Storage Temperature				
	
	-20°C	+4°C	+20°C	+37°C	+45°C
8	0.00	-0.04	0.26	0.41	0.44
12	0.00	0.06	0.35	0.62	0.62

The accelerated thermal degradation samples were stored at +4°C, +20°C, +37°C and 45°C for a period of 8 and 12 months, these samples were compared to vials of AA that were stored continuously at -20°C. After 8 months, six vials of each sample stored at the elevated temperatures were analysed, with all sample extracts being tested singly. After 12 months, two vials of each sample stored at the elevated temperatures were analysed, with all sample extracts being tested duplicate. Mean values are shown for the log_10 _drop in titre.

## Discussion

The use of NAT-based assays for the detection of *P. falciparum *DNA in blood is now being increasingly used in the management and diagnosis of malaria, particularly in countries where travellers are returning from malaria endemic regions. Such NAT-based assays are also being used, on occasion in the primary screening or confirmatory testing of blood used for transfusion [Dr. S. Salueda, personal communication]. In some centres performing vaccine trials, there are reports of using DNA-based assays to monitor parasitaemia using real-time PCR [26]. However there is widespread variation in the sensitivity and specificity of these assays and the aim of this study was to evaluate candidate reference preparations for *P. falciparum *DNA that will be useful for standardization purposes.

The candidate standards were evaluated by a wide range of assays from a large number of laboratories worldwide, as is the requirement of such WHO international collaborative studies [27]. During this study, the participating laboratories used a variety of different extraction and amplification methods for the detection of *P. falciparum *DNA (Table 2). The majority of these assays were developed in-house and therefore it was not unsurprising to see such a spread of results reported. The participating laboratories mainly returned qualitative results for the NAT-based assays, with very few data returned for quantitative assays. As a consequence, some variability was expected in the range of log_10 _PCR-detectable units/ml, and indeed was found to be comparable to that seen in previous studies carried out to establish other International Standard for NAT-based assays for the blood borne viruses such as HCV [13-17]. With the exclusion of outlier results, the ranges seen in the other studies are typically between 1.5 and 2.5 log_10 _PCR-detectable units/ml. Whilst many of assays targeted the 18S rRNA gene, other regions of the *P. falciparum *genome were used for analysis including the small subunit rRNA gene, the β-tubulin gene and the conserved ATS domain of var genes (Table 2). Despite the differences in the genes targeted by the different assays, there is still a wide range of results, as described above, even when the same region of the *P. falciparum *genome is targeted, such as the multicopy 18S rRNA gene. In the case of Laboratory 3A, results were reported for a PCR assay directed against the 18S rRNA gene, which gave comparable results to laboratory 7 targeting the single copy β-tubulin gene. In a recent study [28], comparing PCR-based detection methods for *P. falciparum *on a panel of field samples from Nigeria, a range of assays based upon single or multicopy genes gave a range, the sensitivities of which broadly correlated with copy number, however, this is based upon the work of a single group and not a multicentre study. It would appear that even when the same genes are targeted the difference in response observed by the laboratories will be dependent upon the way the assays are designed (e.g. primer and probe sequences) and implemented (reaction mixes, buffer components, performance characteristics and calibration status of thermal cyclers etc.). The availability of an International Standard will help to reconcile differences such as those mentioned above.

To serve as an International Standard, ideally the preparation should be stable for many years and this is the reason that lyophilized preparations are preferred, where residual moisture and oxygen are minimized [27]. From the Arrhenius model for accelerated degradation [24,25] the estimates of the potency for the samples stored at elevated temperatures can be used to predict the long term stability at various temperatures. As a consequence accelerated thermal degradation studies were carried out. In the initial stability studies performed on the lyophilized candidate AA, it was observed that there was very little degradation at lower temperatures (i.e. +4°C and +20°C). There was some observed degradation to the samples stored at +37°C and +45°C. This may be due to natural degradation expected at these temperatures, but may also be the result of increasing difficulty in resuspending samples stored at elevated temperatures over time. Whilst the results for the stability of candidate AA are not such a good fit with the model, they nevertheless suggest that this preparation is of adequate stability and support the long term use of the material. Moreover, the results of the stability studies show that a short time at elevated temperatures during shipment of the standard at ambient temperatures should not cause unacceptable loss of potency. Studies are continuing in order to monitor the real-time stability of this material.

The discussions outlined above suggest that candidate AA would be the most suitable to serve as the International Standard. It also has the advantage of being based on blood from a naturally occurring *P. falciparum *infection and would therefore most closely simulate the field situation. However it should still be noted that the results for candidate BB, based upon the *P. falciparum *3D7 clone cultured in leucodepleted blood, demonstrated that the potencies of the two preparations were near identical despite their different formulation and that results are commutable for these two preparations. Candidate preparation AA was therefore selected as the International Standard as it demonstrated that the DNA concentration was in a suitable range that would be acceptable for use, it was suitable for use in a wide variety of assays utilized by the study participants and it demonstrated good stability. The preparation was assigned an arbitrary unitage in International Units (IU), unrelated to the level of parasitaemia or DNA copy number. These arbitrary units can be used to compare assays or laboratory performance by the response given for the International Standard.

Since the introduction of the 1^st ^WHO International Standard HCV RNA International Standard ten years ago, the number of quantitative assays available has greatly increased. In a recent study to replace the 2^nd ^WHO International Standard for HCV RNA for NAT-based assays [29] there was a marked increase in the number of quantitative assays and very good agreement with the determinations of the potency of the current standard and the candidate replacements. Such standards can be used for quality control and in the determination of the analytical sensitivity of different testing procedures. They also provide a source of material for assay validation and the production of secondary standards to be used as run controls or working reagents. Indeed the data generated in this present study, produced from a large number of laboratories, using a variety of different assays for *P. falciparum *DNA detection, demonstrate the wide range of responses to the panel of candidate standards and reinforce the need for a common standard to allow inter-laboratory comparisons of both in-house and commercial assay sensitivities by traceability to a "gold standard" with a physical existence.

## Conclusion

In October 2006 the ECBS established this preparation as the 1^st ^WHO International Standard for *Plasmodium falciparum *DNA NAT-based assays. This standard has been assigned a unitage of 10^9 ^IU/ml. (5 × 10^8 ^IU per vial, to be reconstituted in 0.5 ml). The 1^st ^WHO International Standard for *P.falciparum *DNA NAT-based assays is stored at the National Institute for Biological Standards and Control (NIBSC code number 04/176). Details on requesting the standard are available on the NIBSC website.

## Authors' contributions

DJP: Design and coordination of the study; molecular studies of the stability of 04/176. Drafting and preparation of the manuscript. ABH: Statistical analysis. PLC & CS: Facilitated the collection of materials for samples AA, CC and DD. Participated in the design of the study. SAB: Participation in the design and coordination of the study. Critically revising the manuscript.
